# Co-Occurrence of Beauvericin and Enniatins in Edible Vegetable Oil Samples, China

**DOI:** 10.3390/toxins11020100

**Published:** 2019-02-09

**Authors:** Xiaomin Han, Wenjing Xu, Jing Zhang, Jin Xu, Fengqin Li

**Affiliations:** NHC Key Laboratory of Food Safety Risk Assessment, China National Center for Food Safety Risk Assessment, Beijing 100021, China; hanxiaomin@cfsa.net.cn (X.H.); xuwenjing@cfsa.net.cn (W.X.); zhangjing@cfsa.net.cn (J.Z.); xujin@cfsa.net.cn (J.X.)

**Keywords:** edible vegetable oil, beauvericin, enniatins, China, UPLC/ESI-MS/MS

## Abstract

A total of 470 edible vegetable oil samples including peanut, soybean, rapeseed, sesame seed, corn, blend, and others collected from eight provinces of China were analyzed for the concentrations of beauvericin (BEA), enniatin A (ENA), A_1_ (ENA_1_), B (ENB), and B_1_ (ENB_1_) by ultraperformance liquid chromatography/electrospray ionization tandem mass spectrometry (UPLC/ESI-MS/MS). Concentrations of BEA, ENB, and ENB_1_ (average = 5.59 μg/kg, 5.16 μg/kg, and 4.61 μg/kg) in all positive samples were higher than those for ENA and ENA_1_ (average = 0.85 μg/kg and 1.88 μg/kg). Frequencies of BEA and ENNs in all analyzed samples were all higher than 50% with the exception of ENA_1_ (36.6%, 172/470). Levels of BEA and ENNs in all analyzed samples varied based on their sample types and geographical distributions (Kruskal–Wallis test, *p* < 0.05). The soybean and peanut oil samples were found to be more easily contaminated by BEA and ENNs than other oil samples. Concentrations of BEA and ENNs in samples obtained from Heilongjiang, Shandong and Guizhou were higher than those found in samples from other provinces. Besides, frequencies of mycotoxin co-contaminations were high and their co-contamination types also varied by oil types. BEA-ENA-ENA_1_-ENB-ENB_1_ was the most commonly found toxin combination type, almost in one third of the analyzed samples (30%, 141/470). Overall, these results indicate that co-occurrence of BEA and ENNs in analyzed Chinese edible vegetable oil samples is highly common, and it is vital to monitor them, both simultaneously and on a widespread level.

## 1. Introduction

Beauvericin (BEA) and enniatins (ENNs) are emerging cyclic hexadepsipeptides mycotoxins mainly produced by the fungi of *Fusarium* species such as *F. oxysporum*, *F. avenaceum*, and *F. poae* [1,2]. They are structurally related and consisting of three alternating hydroxyisovaleryl and N-methylamino acid residues [1,2]. So far, according to the scientific opinion on the risks to human and animal health related to the presence of BEA and ENNs in food and feed, 29 naturally occurring ENN analogues have been identified, but only four ENNs including enniatin A (ENA), A_1_ (ENA_1_), B (ENB), and B_1_ (ENB_1_) are most frequently detected in various foods and feeds [3].

BEA and ENNs are gaining increasing attention due to their diverse biological activities, which include being toxic to viruses, insects, and fungi [4,5,6,7], or being genotoxic such as inducing chromosomal aberrations, sister-chromatid exchanges, and micronucleus [8]. Recently, cytotoxicities of BEA and ENNs on different human cell lines have been reported, including being the inducers of oxidative stress, and the inhibitors of ionophores and enzymes [9,10,11,12]. Furthermore, BEA and ENNs have been found in many countries such as Morocco [13,14], Spain [15], Tunisia [16], Italy [17], Portugal [18], Poland [19], Norway [20], Finland [21], Iran [22], Sweden [23], and China [24]. The more important is that most of them are focused on cereals such as wheat, barley, maize, and oats, as well as cereal-based food [13,14,15,16,17,18,19,20,21,22,23,24]. High frequencies and concentrations of BEA and ENNs in cereal and cereal-based products have been reported in recent years [13,14,15,16,17,18,19,20,21,22,23,24]. However, to our knowledge, there are no systematic contamination data about the natural occurrence of BEA and ENNs in the edible vegetable oil samples up to now.

Edible vegetable oils extracted from seeds have gained immense popularity over animal-based fats in China, mainly due to their potential therapeutic/health-promoting potential benefits. In this study, seven kinds of edible vegetable oils totaling 470 samples collected from eight provinces of China are used to detect their levels of BEA and ENNs. These vegetable oils are mainly produced from vegetable seeds such as peanut, soybean, rapeseed seed, sesame seed, and corn. Several reports indicate that the majority of the edible oil-yielding seeds can be infected with toxin and toxin-producing fungi during their growth, their processing, and their storage period if the conditions are favorable to fungi growth and toxin production [25,26,27,28]. These toxins can be left over in the edible vegetable oils and enter the human food chain by intake of the edible oils and their related products. Increasing research on mycotoxins in the Chinese edible vegetable oil samples has been carried out over recent decades, including aflatoxins [25,26], ochratoxin A [26], zearalenone and its analogues [27], deoxynivalenol [27], nivalenol [27], T-2 toxin [27], and fumonisins [28], but none have been concerned with BEA and ENNs up to now. Therefore, the aim of this study is to evaluate the natural occurrence of BEA and ENNs in the edible vegetable oils at the national level of China by ultraperformance liquid chromatography/electrospray ionization tandem mass spectrometry (UPLC/ESI-MS/MS).

## 2. Results

### 2.1. Natural Occurrence of BEA and ENNs in All Edible Vegetable Oils of China

**Figure 1:**
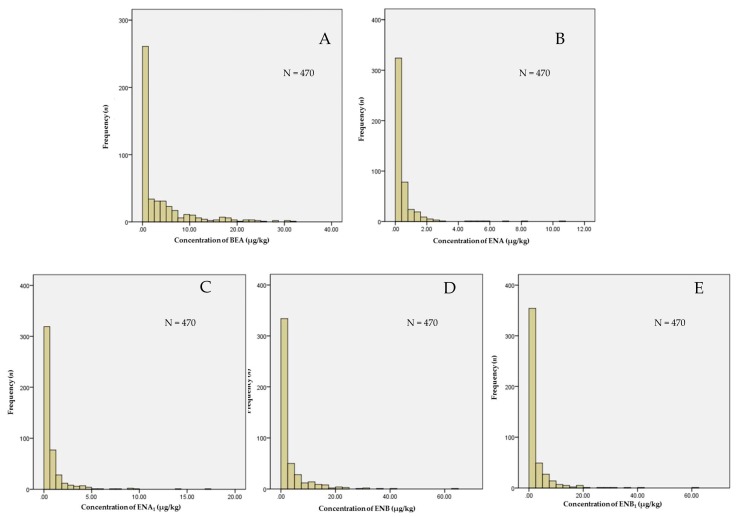
The histogram showing frequency distribution of the natural occurrence of BEA and ENNs in all analyzed samples. BEA = beauvericin, ENNs = enniatins, ENA = enniatin A, ENA_1_ = enniatin A_1_, ENB = enniatin B, ENB_1_ = enniatin B_1_, *n* = sample number, SD = standard deviation. (**A**): The histogram showing frequency distribution of BEA concentrations; (**B**): The histogram showing frequency distribution of ENA concentrations; (**C**): The histogram showing frequency distribution of ENA_1_ concentrations; (**D**): The histogram showing frequency distribution of ENB concentrations; (**E**): The histogram showing frequency distribution of ENB_1_ concentrations.

The histogram showing frequency distribution of the natural occurrence of BEA and ENNs in a total of 470 analyzed oil samples was presented in Figure 1. It was found that they were separated into different groups based on their concentrations, while a large number of samples with the low concentrations aggregated in the left part of Figure 1 and the distribution of the concentrations of BEA and ENNs in all analyzed samples did not follow the normal distribution with the average of 5.59 μg/kg for BEA, 0.85 μg/kg for ENA, 1.88 μg/kg for ENA_1_, 5.16 μg/kg for ENB, and 4.61 μg/kg for ENB_1_ in all positive oil samples, respectively.

The natural occurrence of BEA and ENNs in all analyzed oil samples was given in Table 1. BEA and ENB were the predominant toxins in terms of frequencies and concentrations. It was found that 65.1% (306/470) samples were contaminated by BEA with the concentrations higher than LOQ, followed by ENB with 57.7% (271/470), and ENB_1_ with 50.9% (239/470), and ENA with 50.2% (236/470), and ENA_1_ with 36.6% (172/470). The concentrations of BEA, ENA, ENA_1_, ENB, and ENB_1_ in all analyzed samples varied in oil types (Kruskal–Wallis test, *p* < 0.05). For 98 peanut oil samples, BEA was the predominant toxin based on the frequency and concentration, while four (4.1%) peanut oil samples were contaminated with BEA at the levels higher than 20 μg/kg, 14 (14.3%) between 10 μg/kg and 20 μg/kg, and 82 (83.7%) below 10 μg/kg. Among 133 soybean oil samples analyzed, concentrations of BEA and 4 ENNs were higher than those observed in analyzed positive peanut oil samples with the average concentration trend of BEA > ENB > ENB_1_ > ENA_1_ > ENA, except for ENA_1_. Furthermore, the maximum levels of the five mycotoxins in analyzed 470 samples were all found in soybean oil samples with the concentration of 31.44 μg/kg for BEA, 10.73 μg/kg for ENA, 17.49 μg/kg for ENA_1_, 64.21 μg/kg for ENB, and 61.89 μg/kg for ENB_1_. Besides, 11 (8.3%) soybean oil samples were contaminated with BEA at the levels higher than 20 μg/kg, 20 (15.0%) between 10 μg/kg and 20 μg/kg, and 102 (76.7%) below 10 μg/kg. A total of 6 (4.5%) soybean oil samples were contaminated with ENB at the levels higher than 20 μg/kg, 11 (8.3%) between 10 μg/kg and 20 μg/kg, and 116 (87.2%) below 10 μg/kg. In terms of 100 rapeseed oil samples, ENB was the predominant toxin, and detected in 78.0% (78/100) samples at the levels ranging from 0.50 μg/kg to 40.34 μg/kg (average = 7.81 μg/kg, median = 5.60 μg/kg), which was different for those found in peanut and soybean oil samples. For 35 sesame seed oil samples, only BEA was detected at a high frequency (97.1%, 34/35). For 16 corn oils, only ENB (12.5%, 2/16) was detected in the analyzed corn oil samples. For 30 blend oils and 58 others, the average concentrations of the five mycotoxins in those positive samples were all lower than 2.00 μg/kg, but not for ENA (average = 2.14 μg/kg), ENA_1_ (average = 4.63 μg/kg), and ENB_1_ (average = 2.64 μg/kg) in positive blend oils, and the details were shown in Table 1.

### 2.2. Natural Occurrence of BEA and ENNs in All Edible Vegetable Oils From Different Provinces of China

The natural occurrence of the five mycotoxins in all analyzed samples from different provinces of China was presented in Table 2, and the concentrations of the five mycotoxins were significantly different in samples collected from different provinces (Kruskal–Wallis test, *p* < 0.05). In terms of BEA, the concentrations of BEA in samples from Heilongjiang (average = 8.14 μg/kg) and Shandong (average = 7.15 μg/kg) were 1.07 to 4.02 times higher than those from other provinces. Frequencies of BEA in samples from Heilongjiang (76.2%, 64/84), Shandong (82.8%, 130/157), and Guizhou (75.9%, 22/29) were higher for than those found in samples from Hebei (52.4%, 11/21), Jiangsu (38.0%, 19/50), Sichuan (49.4%, 39/79), Yunnan (53.8%, 7/13), and Guangxi (37.8%, 14/37). For ENA and ENA_1_, the average concentrations of ENA in samples from different provinces were all below or close to the concentration of 1.00 μg/kg, but not for ENA in samples from Guizhou; while for ENA_1_, the average concentrations of ENA_1_ in samples from different provinces were all higher than 2.00 μg/kg with the exception of ENA_1_ in Jiangsu, Sichuan, and Guangxi. For ENB and ENB_1_, the average concentrations of ENB and ENB_1_ in samples from different provinces were higher than 1.00 μg/kg, with the maximum of 11.96 μg/kg for ENB and 7.37 μg/kg for ENB_1_ both in Guizhou. In sum, the oil samples from Heilongjiang, Shandong, and Guizhou were more easily contaminated by the five mycotoxins than the samples from other provinces.

### 2.3. Contamination and Co-contamination of BEA and ENNs in All Edible Vegetable Oils in China

Contamination and co-contamination of the five mycotoxins in all analyzed oil samples were shown in Table 3. It was found that 95 (20%, 95/470) and 81 (17%, 81/470) samples were not contaminated by any kind of the five mycotoxins and contaminated by only one mycotoxin, respectively, while 294 (63%, 294/470) samples were contaminated by at least two mycotoxins. The most common combination was BEA-ENA-ENA_1_-ENB-ENB_1_ with 141 (30%, 141/470) samples including 6 (6%, 6/98) peanut oils, 82 (62%, 82/133) soybean oils, 51 (51%, 51/100) rapeseed oils, one (3%, 1/35) sesame seed oils, and one (2%, 1/58) other oils, which were almost one third of the analyzed samples, followed by 67 (14%, 14/470) samples co-contaminated by the two mycotoxins, 46 (10%, 46/470) by the four mycotoxins, and 40 (9%, 40/470) by the three mycotoxins, respectively.

The co-contamination types of the five mycotoxins in the analyzed samples varied by oil types. BEA-ENA-ENA_1_-ENB-ENB_1_ was also the most frequent toxin combination in soybean oils (62%, 82/133) and rapeseed oils (51%, 51/100), followed by the two mycotoxins co-contamination in soybean oils (5%, 7/133) and rapeseed oils (18%, 18/100); while for peanut oils, sesame seed oils, blend oils, and others, the most frequent toxin co-contamination type was the two mycotoxins co-contamination with the frequency of 24% (24/98) in peanuts oils, 23% (8/35) in sesame seed oils, 40% (12/30) in blend oils, and 22% (13/58) in others, and the second frequent toxin co-contamination type in the above four oil types was all the three mycotoxins co-contamination with 19% in peanut oils, 6% in sesame seed oils, 7% in blend oils, and 9% in others. However, for corn oils, no sample was contaminated with two or more kinds of mycotoxins simultaneously, and there were only two contamination types including 88% (14/16) samples not contaminated by any kind of the five mycotoxins and 12% (2/16) samples contaminated by only one mycotoxin.

## 3. Discussion

China, as the country with the largest population and the greatest consumption of edible oil in the world, has a strong demand for edible vegetable oils. The annual per-capita for edible vegetable oil was increasing year by year, and increased from 2.20 kg per year in the 1980s to higher than 11.70 kg per year in 2014 [29,30]. Therefore, it is very important to ensure the safety of edible oils. As we know, in China, vegetable oils are often made from seeds such as peanut, soybean, rapeseed, and others, which may be contaminated by mycotoxins and mycotoxin-producing fungi, including BEA and ENNs and these toxins producing fungi *Fusarium* species [31,32,33,34,35,36]. Although the maximum levels and the related national standard determination method for BEA and ENNs have not been established, their toxicity in combination with high contamination levels could pose possible hazards to health. The European Commission has requested for EFSA opinion on the risks to human and animal health related to BEA and ENNs in food and feed [3]. However, to the best of our knowledge, this is the first report on the natural occurrence of BEA and ENNs in Chinese edible vegetable oils.

One interesting founding was that BEA and ENNs were found as the co-contaminations in the analyzed edible vegetable oil samples. Out of the total samples, 294 (63%, 294/470) samples were contaminated by at least two mycotoxins including 141 (30%, 141/470) samples contaminated by the five mycotoxins simultaneously. The reason might be that BEA and ENNs producing fungi *Fusarium* species such as *F. equiseti*, *F. oxysporum*, and *F. avenaceum* could infect the seeds used for oil production simultaneously [31,32,33,34,35,36]. The second interesting founding was that the levels of BEA and ENNs in those edible vegetable oil samples were lower than those found in cereal samples, which did not undergo further processing such as cooking and heating [13,14,15,16,17,18,19,20,21,22,23]. Most of the oil samples analyzed were collected from the big companies and needed to undergo several processing processes. The squeezing processing and the leaching technology were the two popular technologies used in the Chinese oil industry [37], both of which needed to undergo the cooking process at a high temperature (80 °C). Besides, Serrano et al. reported that the temperature facilitated a marked reduction in ENNs content during the white and whole-grain pasta processing, and up to 50% and 80% reduction of ENNs was achieved by drying pasta at 45 °C–55 °C and 70 °C–90 °C, respectively, but not for low temperature (25 °C) [38].

Furthermore, it was found that the concentrations of BEA and ENNs in all analyzed samples varied by oil types and collection provinces. Samples such as soybean and peanut oils, and samples from Heilongjiang, Shandong, and Guizhou were more easily contaminated than others. The reason for this may due to the difference of the community ecology of fungal pathogens between different seeds and different provinces. Xu and Nicholson reported that the cooler environment was more suitable for the growth of *Fusarium* species including *F.culmorum* and *F.avenaceum* [39], which may indicated that samples from the cooler district could be contaminated with high levels of BEA and ENNs. However, to date, limited information was available on the natural occurrence of BEA and ENNs and the toxin producing fungi species in seeds used for oil production.

In conclusion, we reported for the first time about the natural occurrence of emerging *Fusarium* mycotoxins BEA and ENNs in Chinese edible vegetable oil samples. We found that the co-contamination of BEA and ENNs was common in studied oils and the levels of BEA and ENNs varied by oil types and geographical distributions. Although the acceptable daily intake (ADI) and the maximum levels are not established, it is necessary to conduct the detailed BEA and ENNs monitoring programs in China based on their high frequencies and co-contaminations.

## 4. Materials and Methods

### 4.1. Chemicals and Reagents

Standard solid powers of BEA, ENA, ENA_1_, ENB, and ENB_1_ with purity ≥97% were purchased from Bioaustralis (Smithfield, Australia). Acetonitrile (ACN) and methanol (MeOH), both of LC-MS grade, were obtained from Fisher Scientific (Fair Lawn, NJ, USA). Ammonium acetate was in MS grade from Sigma-Aldrich (St. Louis, MO, USA). Water was purified successively by reverse osmosis and a Millipore Milli-Q system (Millipore, Bedford, MA, USA) with a conductivity ≥18.2 MΩ.cm at 25 °C.

### 4.2. Samples Collection

A total of 470 edible vegetable oil samples including peanut oil, soybean oil, rapeseed oil, sesame seed oil, corn oil, blend oil samples and others were randomly collected by visiting supermarkets and agricultural trade markets. They were obtained from the most important edible vegetable oil-producing regions including Heilongjiang, Hebei, Shandong, Jiangsu, Guizhou, Sichuan, Yunnan, and Guangxi, and their yearly edible vegetable oil output accounted for about 70% of the total in China. Most of the oil samples analyzed are collected from the big companies which can be sold both at home and abroad, therefore several processing processes including the squeezing processing and the leaching technology are applied in oil production. All samples were kept at 4 °C until analysis. Each sample was mixed thoroughly before taking and 5 g test portion was taken for analysis.

### 4.3. UPLC Conditions

UPLC/ESI-MS/MS system equipped with ExionLC (SHIMADZU, Kyoto, Japan), QTRAP^TM^ 5500 MS/MS system (AB Sciex, Foster City, CA, USA) and a MultiQuant^TM^ Version 3.0.2 software (AB Sciex, Foster City, CA, USA) for data acquisition and analysis was used to quantify the five mycotoxins in the positive mode. Chromatographic separation was achieved using a C_18_ column (2.1 mm × 50 mm, 1.7 μm bead diameters, Waters, Milford, MA, USA). Temperatures of the UPLC column and autosampler were set at 35 °C and 15 °C, respectively. The gradient elution program included the mobile phase A (containing 2 mmol/L ammonium acetate) and the mobile phase B (acetonitrile) with a flow rate of 0.2 mL/min. The detailed information was shown in Table 4.

### 4.4. MS Conditions

Multiple reaction monitoring (MRM) was used for data acquisition with an optimized dwell time of 200 ms in order to obtain the optimal sensitivity and selectivity of MS conditions in positive electrospray ionization (ESI^+^) mode, and the ion source parameters for BEA and ENNs determination were shown in Table 5. Parent and fragment ions (quantifier and qualifier) for each analyte were chosen regarding the best signal-to-noise ratios in a spiked blank sample [40]. The MRM parameters for BEA and ENNs detection were shown in Supplementary Table S1.

### 4.5. Extraction and Analysis of BEA and ENNs

The extraction method of BEA, ENA, ENA_1_, ENB, and ENB_1_ from edible vegetable oil was modified on the basis of the methods developed previously [40]. Because all the edible vegetable oils were in liquid form, five grams of each oil sample were extracted with 40 mL of acetonitrile-water (85:15, vol/vol) by blending 30–60 s using vortex and then shaking for 30 min. The extract was transferred to a new 50 mL centrifuge tube and allowed standing for 15–20 min under room temperature. Each aliquot of 10 mL of supernatant was diluted with 20 mL of distilled water and the diluted extract was homogeneously blended using vortex. Four mL of the diluted extract was applied to a Sep-Pak Vac 3cc (200 mg) C_18_ Cartridge (Waters, Milford, MA, USA), which was pre-equilibrated with 3 mL of methanol and 3 mL of water. The cartridge was washed with 3 mL of 10% acetonitrile in water followed by a wash with 3 mL of 50% acetonitrile in water. Toxins were eluted using 2.0 mL of 90% acetonitrile in water. Matrix-matched calibration was used for quantification of BEA and ENNs in edible vegetable oils. Mean recoveries, in which the matrix effect was compensated for, were in the ranges of 82.9–115.5% (BEA), 70.1–110.7% (ENA), 71.8–101.2% (ENA_1_), 78.8–102.9% (ENB), and 78.8–102.9% (ENB_1_) as determined from six parallel analyses of blank vegetable oil samples spiked with 5–25 μg/kg (BEA), 20–100 μg/kg (ENA), 1–5 μg/kg (ENA_1_), 1–5 μg/kg (ENB) and 0.25–1.25 μg/kg (ENB_1_) with the respective coefficient of variation (CV) of 9.9–13.1% for BEA, 4.4–16.5% for ENA, 10.7–12.3% for ENA_1_, 10.7–12.3% for ENB, and 6.6–12.4% for ENB_1_, respectively. All the regressive equations, correlation coefficient and the limits of detection and limits of quantification for BEA, ENA, ENA_1_, ENB, and ENB_1_ in different edible vegetable oils were presented in Table 6.

### 4.6. Data Analysis

The SPSS statistical package (version 19.0, IBM, Amund, NY, USA) was applied for the calculation of all parameters including the positive rate, the average, the median, the range, and the Kruskal–Wallis test of BEA and ENNs in the analyzed samples. Kruskal–Wallis test was employed for statistical analysis in comparison of the mycotoxin concentrations in different edible vegetable oil samples.

## Figures and Tables

**Table 1:** Natural occurrence of BEA and ENNs in all edible vegetable oils in China (*n* = 470).

Sample	Mycotoxin ^a^	(≥LOQ ^b^) % (*n*)	Range (μg/kg)	Average (μg/kg)	Median (μg/kg)	SD ^c^
Peanut oil (*n* = 98)	BEA	78.6 (77)	1.31–30.42	7.29	5.23	6.12
	ENA	55.1 (54)	0.33–2.96	0.89	0.73	0.57
	ENA_1_	8.2 (8)	2.15–4.74	3.29	3.16	1.02
	ENB	42.9 (42)	0.26–19.87	2.73	1.34	3.80
	ENB_1_	41.8 (41)	0.63–18.70	3.36	1.94	4.12
Soybean oil (*n* = 133)	BEA	72.2 (96)	0.22–31.44	9.20	6.34	7.86
	ENA	66.9 (89)	0.14–10.73	0.93	0.38	1.47
	ENA_1_	63.2 (84)	0.46–17.49	2.12	1.17	2.44
	ENB	73.7 (98)	0.13–64.21	6.28	3.10	8.83
	ENB_1_	70.7 (94)	0.23–61.89	6.27	2.90	8.83
Rapeseed oil (*n* = 100)	BEA	64.0(64)	0.16–11.57	1.40	0.53	1.97
	ENA	83.0(83)	0.27–8.09	0.76	0.45	1.22
	ENA _1_	69.0 (69)	0.51–14.2	1.44	0.96	1.95
	ENB	78.0 (78)	0.50–40.34	7.81	5.60	7.71
	ENB_1_	76.0 (76)	0.09–42.41	4.18	2.53	5.87
Sesame seed oil (*n* = 35)	BEA	97.1 (34)	0.33–16.79	4.52	3.11	4.32
	ENA	14.3 (5)	0.33–0.54	0.41	0.37	0.09
	ENA_1_	2.9 (1)	0.87–0.87	0.87	0.87	no ^e^
	ENB	22.9 (8)	0.12–5.22	0.92	0.25	1.75
	ENB_1_	5.7 (2)	0.54–2.93	1.74	1.74	1.69
Corn oil (*n* = 16)	BEA	nd ^d^	nd	nd	nd	no
	ENA	nd	nd	nd	nd	no
	ENA_1_	nd	nd	nd	nd	no
	ENB	12.5 (2)	0.23–0.52	0.37	0.37	0.20
	ENB_1_	nd	nd	nd	nd	no
Blend oil (*n* = 30)	BEA	50.0 (15)	0.20–6.57	0.81	0.36	1.61
	ENA	3.3 (1)	2.14–2.14	2.14	2.14	no ^e^
	ENA_1_	6.7 (2)	0.41–8.85	4.63	4.63	5.97
	ENB	56.7 (17)	0.05–16.34	1.29	0.20	3.90
	ENB_1_	33.3 (10)	0.17–22.16	2.64	0.45	6.87
Others (*n* = 58)	BEA	34.5 (20)	0.18–1.69	0.54	0.34	0.44
	ENA	6.9 (4)	0.56–1.23	0.86	0.82	0.29
	ENA_1_	13.8 (8)	0.37–2.81	1.27	1.24	0.78
	ENB	44.8 (26)	0.05–4.59	1.12	0.50	1.34
	ENB_1_	27.6 (16)	0.20–6.40	1.80	0.89	1.88
Total (*n* = 470)	BEA	65.1 (306)	0.16–31.44	5.59	3.34	6.55
	ENA	50.2 (236)	0.14–10.73	0.85	0.48	1.19
	ENA_1_	36.6 (172)	0.37–17.49	1.88	1.07	2.24
	ENB	57.7 (271)	0.05–64.21	5.16	2.51	7.41
	ENB_1_	50.9 (239)	0.09–61.89	4.61	2.36	6.95

^a^: BEA = beauvericin, ENA = enniatin A, ENA_1_ = enniatin A_1_, ENB = enniatin B, ENB_1_ = enniatin B_1_; ^b^: LOQ = limit of quantification; ^c^: SD = standard deviation; ^d^: nd = not detected i.e., less than the limit of the quantification (<LOQ); ^e^: no = without SD; *n* = number of samples.

**Table 2:** Natural occurrence of BEA and ENNs in all edible vegetable oils from different provinces (*n* = 470).

Province	Mycotoxin	(≥LOQ) % (*n*)	Range (μg/kg)	Average (μg/kg)	Median (μg/kg)	SD
Heilongjiang (*n* = 84)	BEA	76.2 (64)	0.25–31.2	8.14	6.06	7.13
	ENA	71.4 (60)	0.14–10.7	1.06	0.34	1.75
	ENA_1_	65.5 (55)	0.46–17.5	2.03	0.99	2.69
	ENB	82.1 (69)	0.09–64.2	5.75	2.29	9.56
	ENB_1_	75.0 (63)	0.30–61.9	5.59	2.20	9.21
Hebei (*n* = 21)	BEA	52.4 (11)	0.26–6.0	2.35	1.54	2.04
	ENA	23.8 (5)	0.29–1.4	1.01	1.35	0.53
	ENA_1_	19.0 (4)	0.91–3.2	2.44	2.84	1.07
	ENB	47.6 (10)	0.05–7.1	2.59	0.88	2.86
	ENB_1_	28.6 (6)	0.52–8.3	4.55	4.41	3.29
Shandong (*n* = 157)	BEA	82.8 (130)	0.18–31.4	7.15	4.44	7.17
	ENA	47.1 (74)	0.17–3.0	0.81	0.67	0.55
	ENA_1_	23.6 (37)	0.50–8.9	2.40	1.78	1.83
	ENB	51.0 (80)	0.05–31.4	3.97	2.02	5.62
	ENB_1_	43.3 (68)	0.17–35.3	5.15	2.95	6.59
Jiangsu (*n* = 50)	BEA	38.0 (19)	0.21–23.3	3.46	0.49	6.20
	ENA	26.0 (13)	0.23–1.4	0.46	0.38	0.29
	ENA_1_	24.0 (12)	0.58–1.4	0.89	0.90	0.19
	ENB	46.0 (23)	0.06–14.0	3.51	1.71	4.19
	ENB_1_	38.0 (19)	0.21–6.5	2.17	1.54	1.82
Guizhou (*n* = 29)	BEA	75.9 (22)	0.17–4.5	1.91	1.50	1.51
	ENA	89.7 (26)	0.30–8.1	1.40	0.65	2.03
	ENA_1_	79.3 (23)	0.55–14.2	2.44	1.56	3.16
	ENB	86.2 (25)	0.73–40.3	11.96	10.32	9.77
	ENB_1_	86.2 (25)	0.34–42.4	7.37	5.22	8.98
Sichuan (*n* = 79)	BEA	49.4 (39)	0.16–15.9	1.62	0.43	2.97
	ENA	53.2 (42)	0.27–1.1	0.43	0.40	0.17
	ENA_1_	41.8 (33)	0.37–2.3	0.93	0.83	0.38
	ENB	60.8 (48)	0.10–23.2	4.37	2.77	5.18
	ENB_1_	54.4 (43)	0.09–9.0	2.16	1.48	2.23
Yunnan (*n* = 13)	BEA	53.8 (7)	0.17–11.6	2.94	0.55	4.15
	ENA	61.5 (8)	0.46–2.1	1.01	0.86	0.57
	ENA_1_	53.8 (7)	0.67–8.9	2.27	1.27	2.92
	ENB	53.8 (7)	0.08–21.1	8.41	4.61	8.38
	ENB_1_	53.8 (7)	0.38–22.2	5.80	2.32	7.79
Guangxi (*n* = 37)	BEA	37.8 (14)	0.26–7.1	3.05	2.38	1.98
	ENA	21.6 (8)	0.33–0.7	0.50	0.46	0.14
	ENA_1_	2.7 (1)	0.41–1.1	0.41	0.41	no
	ENB	24.3 (9)	0.11–2.0	1.02	1.00	0.71
	ENB_1_	21.6 (8)	0.63–3.0	1.77	1.84	0.74

**Table 3:** Contamination and co-contamination of the five mycotoxins in all edible vegetable oil samples from parts of China (*n* = 470).

Contamination by	Frequency % (*n*) for Mycotoxin Contamination and Co-contamination								
	Peanut Oil (*n* = 98)	Soybean Oil (*n* = 133)	Rapeseed Oil (*n* = 100)	Sesame Seed Oil (*n* = 35)	Corn Oil (*n* = 16)	Blend Oil (*n* = 30)	Others (*n* = 58)	Sum1 (*n* = 470)	Sum (*n* = 470)
ND	13 (13)	25 (33)	10 (10)	3 (1)	88(14)	13 (4)	34 (20)	20 (95)	20 (95)
One mycotoxin	19 (19)	3 (4)	6 (6)	66 (23)	12 (2)	37 (11)	28 (16)	17 (81)	17(81)
Two mycotoxins	24 (24)	2 (4)	8 (8)	23 (8)	0 (0)	40 (12)	22 (13)	14 (67)	63 (294)
Three mycotoxins	19 (19)	4 (5)	7 (7)	6 (2)	0 (0)	7 (2)	9 (5)	9 (40)	
Four mycotoxins	17 (17)	5 (7)	18 (18)	0 (0)	0 (0)	3 (1)	5 (3)	10 (46)	
Five mycotoxins	6 (6)	62 (82)	51 (51)	3 (1)	0 (0)	0 (0)	2 (1)	30 (141)	

ND = not contaminated by any kind of the five mycotoxins studied.

**Table 4:** The gradient elution program for BEA and ENNs detection.

Time (min)	Mobile Phase A (%)	Mobile Phase B (%)
0	100	0
2	100	0
3	40	60
19	30	70
21	100	0
21.1	100	0

**Table 5:** Ion source parameters for BEA and ENNs detection.

Parameter	Character
Curtain gas	30 psi
Collision gas	medium
Ion spray voltage	5500 v
Source desolvation temperature	550 °C
Gas 1	80 psi
Gas 2	80 psi

**Table 6:** Parameters associated with the calibration curve and method sensitivity for the detection of BEA and ENNs for different oils.

Sample	Mycotoxin	Regressive Equation	Correlation Coefficient	LOD (μg/kg)	LOQ (μg/kg)
Peanut oil	BEA	*y* = 1831910 *x* + 658263	0.99334	0.29	1.30
	ENA	*y* = 4016180 *x* − 166542	0.99371	0.09	0.31
	ENA_1_	*y* = 2543860 *x* − 60391.4	0.99367	0.43	1.78
	ENB	*y* = 3020570 *x* + 437387	0.99333	0.08	0.26
	ENB_1_	*y* = 1627330 *x* + 1130430	0.99267	0.18	0.61
Soybean oil	BEA	*y* = 183690 *x* + 510190	0.99862	0.29	1.30
	ENA	*y* = 3411850 *x* + 90468.4	0.99919	0.05	0.16
	ENA_1_	*y* = 2505060 *x* + 217001	0.99913	0.04	0.13
	ENB	*y* = 3206470 *x* + 369942	0.99889	0.13	0.43
	ENB_1_	*y* = 1635060 *x* + 477786	0.99873	0.04	0.12
Rapeseed oil	BEA	*y* = 266531 *x* + 3185.30194	0.99979	0.04	0.14
	ENA	*y* = 591485 *x* + 9695.51985	0.99550	0.08	0.27
	ENA_1_	*y* = 469491 *x* + 9159.34417	0.99973	0.15	0.5
	ENB	*y* = 651794 *x* + 6229.89274	0.99969	0.14	0.48
	ENB_1_	*y* = 610548 *x* + 1769.24413	1.00000	0.02	0.07
Sesame seed oil	BEA	*y* = 258595 *x* + 6810.67455	0.99969	0.09	0.29
	ENA	*y* = 676118 *x* + 6329.11448	1.00000	0.09	0.31
	ENA_1_	*y* = 548739 *x* + 3559.07382	1.00000	0.12	0.39
	ENB	*y* = 387395 *x* + 3820.26566	1.00000	0.03	0.11
	ENB_1_	*y* = 275432 *x* + 3922.63450	0.99972	0.06	0.19
Corn oil	BEA	*y* = 265163 *x* + 2844.56953	0.99998	0.21	0.7
	ENA	*y* =588296 *x* + 1879.55578	0.99975	0.16	0.53
	ENA_1_	*y* = 459591 *x* + 177.44785	0.99988	0.38	1.26
	ENB	*y* = 610548 *x* + 1760.24413	1.00000	0.07	0.23
	ENB_1_	*y* = 728990 *x* + 1788.38369	0.99997	0.36	1.21
Blend oil	BEA	*y* = 268840 *x* + 472.32478	0.99986	0.06	0.18
	ENA	*y* = 599369 *x* + 2058.81347	0.99984	0.16	0.52
	ENA_1_	*y* = 449363 *x* + 1376.66349	0.99965	0.11	0.35
	ENB	*y* = 603225 *x* + 2395.36332	0.99990	0.02	0.05
	ENB_1_	*y* = 268600 *x* + 778.00995	0.99959	0.05	0.16

## Supplementary Materials

The following are available online at https://www.mdpi.com/2072-6651/11/2/100/s1, Table S1: The MRM parameters of MS/MS conditions for BEA and ENNs detection.

## References

[1] Hamill R.L., Higgens C.E., Boaz H.E., Gorman M. (1969). The structure of beauvericin, a new depsipeptide antibiotic toxic to *Artemia salina*. Tetrahedron Lett..

[2] Blais L.A., Apsimon J.W., Blackwell B.A., Greenhalgh R., Miller J.D. (1992). Isolation and characterization of enniatins from *Fusarium avenaceum* DAOM 196490. Can. J. Chem..

[3] European Food Scientific Authority Panel on Contaminants in the Food Chain (CONTAM) (2016). Scientific opinion on the risks to human and animal health related to the presence of beauvericin and enniatins in food and feed. EFSA J..

[4] Shin C.-G., An D.-G., Song H.-H., Lee C. (2009). Beavericin and enniatins H, I and MK1688 are new potent inhibitors of human immunodeficiency virus type-1 integrase. J. Antibiot..

[5] Gupta S., Krasnoff S.B., Underwood N.L., Renwick J.A., Roberts D.W. (1991). Isolation of beauvericin as an insect toxin from *Fusarium semitectum* and *Fusarium moniliforme* var. *subglutinans*. Mycopathologia.

[6] Meca G., Soriano J.M., Gaspari A., Ritieni A., Moretti A., Mañes J. (2010). Antifungal effects of the bioactive compounds enniatins A, A_1_, B, B_1_. Toxicon.

[7] Wang Q.G., Xu L.J. (2012). Beauvericin, a bioactive compound produced by fungi: A short review. Molecules.

[8] Celik M., Aksoy H., Yilmaz S. (2010). Evaluation of beauvericin genotoxicity with the chromosomal aberrations, sister-chromatid exchanges and micronucleus assays. Ecotoxicol. Environ. Safty.

[9] Kamyar M., Rawnduzi P., Studenik C.R., Kouri K., Lemmens-Gruber R. (2004). Investigation of the electrophysiological properties of enniatins. Arch. Biochem. Biophys..

[10] Juan-García A., Ruiz M.J., Font G., Manyes L. (2015). Enniatin A_1_, enniatin B_1_ and beauvericin on HepG2: Evaluation of toxic effects. Food Chem. Toxicol..

[11] Prosperini A., Juan-García A., Font G., Ruiz M.J. (2013). Reactive oxygen species involvement in apoptosis and mitochondrial damage in Caco-2 cells induced by enniatins A, A_1_, B and B_1_. Toxicol. Lett..

[12] Prosperini A., Juan-García A., Font G., Ruiz M.J. (2013). Beauvericin-induced cytotoxicity via ROS production and mitochondrial damage in Caco-2 cells. Toxicol. Lett..

[13] Mahnine N., Meca G., Elabidi A., Fekhaoui M., Saoiabi A., Font G., Mañes J., Zinedine A. (2011). Further data on the levels of emerging Fusarium mycotoxins enniatins (A, A_1_, B, B_1_), beauvericin and fusaproliferin in breakfast and infant cereals from Morocco. Food Chem..

[14] Sifou A., Meca G., Serrano A.B., Mahnine N., Abidi A.E., Mañes J., Azzouzi M.E.A., Zinedine A. (2011). First report on the presence of emerging *Fusarium* mycotoxins enniatins (A, A_1_, B, B_1_), beauvericin and fusaproliferin in rice on the Moroccan retail markets. Food Control.

[15] Meca G., Zinedine A., Blesa J., Font G., Mañes J. (2010). Further data on the presence of *Fusarium* emerging mycotoxins enniatins, fusaproliferin and beauvericin in cereals available on the Spanish markets. Food Chem. Toxicol..

[16] Oueslati S., Meca G., Mliki A., Ghorbel A., Mañes J. (2011). Determination of *Fusarium* mycotoxins enniatins, beauvericin and fusaproliferin in cereals and derived products from Tunisia. Food Control.

[17] Jestoi M., Somma M.C., Kouva M., Veijalainen P., Rizzo A., Ritieni A., Peltonen K. (2004). Levels of mycotoxins and sample cytotoxicity of selected organic and conventional grain-based products purchased from Finnish and Italian markets. Mol. Nutr. Food Res..

[18] Blesa J., Marín R., Lino C.M., Mañes J. (2012). Evaluation of enniatins A, A_1_, B, B_1_ and beauvericin in Portuguese cereal-based foods. Food Addit. Contam. Part A..

[19] Stępień Ł., Gromadzka K., Chełkowski J. (2012). Polymorphism of mycotoxin biosynthetic genes among *Fusarium equiseti* isolates from Italy and Poland. J. Appl. Genet..

[20] Uhlig S., Torp M., Heier B.T. (2006). Beauvericin and enniatins A, A_1_, B and B_1_ in Norwegian grain: A survey. Food Chem..

[21] Jestoi M.N., Paavanen-Huhtala S., Parikka P., Yli-Mattila T. (2008). In vitro and in vivo mycotoxin production of *Fusarium* species isolated from Finnish grains. Arch. Phytopathol. Plant Prot..

[22] Nazari F., Sulyok M., Kobarfard F., Yazdanpanah H., Krska R. (2015). Evaluation of Emerging *Fusarium* mycotoxins beauvericin, Enniatins, Fusaproliferin and Moniliformin in Domestic Rice in Iran. Iran. J. Pharm. Res..

[23] Lindblad M., Gidlund A., Sulyok M., Börjesson T., Krska R., Olsen M., Fredlund E. (2013). Deoxynivalenol and other selected *Fusarium* toxins in Swedish wheat - coccurrence and correlation to specific *Fusarium* species. Int. J. Food Microbiol..

[24] Han X.M., Xu W.J., Zhang J., Xu J., Li F.Q. (2019). Natural occurrence of beauvericin and enniatins in corn- and wheat-based samples harvested in 2017 collected from Shandong province, China. Toxins.

[25] Bao L., Trucksess M.W., White K.D. (2010). Determination of aflatoxin B_1_, B_2_, G_1_, and G_2_ in olive oil, peanut oil, and sesame oil. J. AOAC Int..

[26] Ferrracane R., Tafuri A., Logieco A., Galvano F., Balzano D., Ritieni A. (2007). Simultaneous determination of aflatoxin B_1_ and ochratoxin A and their natural occurrence in Mediterranean virgin olive oil. Food Addit. Contam..

[27] Schollenberger M., Müller H.M., Rüfle M., Drochner W. (2008). Natural occurrence of 16 *Fusarium* toxins in edible oil marketed in Germany. Food Control.

[28] Li F., Jiang D., Zheng F., Chen J., Li W. (2015). Fumonisins B_1_, B_2_ and B_3_ in corn products, wheat flour and corn oil marketed in Shandong province of China. Food Addit. Contam. Part B..

[29] Wang R.Y. (2017). The present research status and the development trend of the edible vegetable oil processing industry in China. Sci. Technol. Cereals Oils Foods.

[30] Wang R.Y. (2018). Thoughts on the new development of China’s grain and oil processing industry in the new era. Mod. Flour Milling Ind..

[31] Bhat R., Reddy K.R.N. (2016). Challenges and issues concerning mycotoxins contamination in oil seeds and their edible oils: updates from last decade. Food Chem..

[32] Kaèergius A., Lugauskas A., Levinskaito L., Mankevikaito A. (2005). Screening of micromycetes producing toxic substances under various conditions. Bot. Lith..

[33] Schollenberger M., Müller H.M., Rüfle M., Suchy S., Plank S., Drochner W. (2005). Survey of Fusarium toxins in foodstuffs of plant origin marketed in Germany. Int. J. Food Microbiol..

[34] Sharfun N.M., Mushtaq M., Hashmi M.H. (2005). Seed-bore mycoflora of sunflower (*Helianthus annuus L.*). Pak. J. Bot..

[35] Khan T., Khan K., Haq M. (2005). Effect of storage fungi on the seed quality parameters of different mustard varieties. Pak. J. Sci. Ind. Res..

[36] Trung T.S., Tabuc C., Bailly S., Querin A., Guerre P., Bailly J.D. (2008). Fungal mycoflora and contamination of maize from Vietnam with aflatoxin B_1_ and fumonision B_1_. World Mycotoxin J..

[37] Sun Z. (2017). The processing technology of the edible vegetable oil. China Food Saf..

[38] Serrano A.B., Font G., Mañes J., Ferrer E. (2016). Effects of technological processes on enniatin levels in pasta. J. Sci. Food Agric..

[39] Xu X., Nicholson P. (2009). Community ecology of fungal pathogens causing wheat head blight. Annu. Rev. Phytopathol..

[40] Han X.-M., Xu W.-J., Zhao X., Zhang H.-Y., Zhang J., Li F.-Q. (2017). Development of high performance liquid chromatography tandem-mass spectrometry method for determination of beauvericin and enniatins in corn and wheat and their products. Chin. J. Food Hyg..

