# Supplementary material for: Co-Occurrence of Beauvericin and Enniatins in Edible Vegetable Oil Samples, China
Source: Toxins (Basel). 2019 Feb 9;11(2):100. doi: 10.3390/toxins11020100 (PMC6409968; doi:10.3390/toxins11020100)
Supplement: Supplementary file 1 [file toxins-11-00100-s001.pdf]

Supplementary Materials: Co-Occurrence of Beauvericin and Enniatins in Edible Vegetable Oil Samples, China

Xiaomin Han, Wenjing Xu, Jing Zhang, Jin Xu and Fengqin Li

Table 1. MRM parameters of MS/MS conditions for BEA and ENNs detection.

Mycotoxin [a]	Parent ion	Daughter Ion	DP [b] (v)	CE [c] (v)	CXP [d] (v)	EP [e] (v)
BEA	801.4	244.1	100	40	12	7
	801.4	262.1	100	40	12	7
ENA	699.5	210.1	100	40	10	7
	699.5	228.2	100	40	12	7
ENA_1	685.5	210.1	84	38	7	7
	685.5	228.2	84	38	7	7
ENB	657.5	196.2	90	38	9	7
	657.5	214.1	90	38	10	7
ENB_1	671.5	196.1	88	40	8	7
	671.5	210.2	88	40	8	7

[a]: BEA = beauvericin, ENA = enniatin A, ENA_1 = enniatin A_1, ENB = enniatin B, ENB_1 = enniatin B_1; [b]: DP = declustering potential; [c]: CE = collision energy; [d]: CXP = collision cell exit potential; [e]: EP = entrance potential.
